# Long-Term DHI Outcomes in Patients with Chronic Dizziness and Initially Uncertain Diagnosis: A Retrospective Follow-Up Study

**DOI:** 10.3390/audiolres16040107

**Published:** 2026-07-28

**Authors:** Johanna Müller-Diesing, Thien An Duong Dinh, Stephan Hackenberg, Anna Klein, Ariane Renson, Justus Ilgner, Julia Kristin Brach, Markus Wirth, Flurin Müller-Diesing

**Affiliations:** 1 Department of Otorhinolaryngology, RWTH Aachen University Hospital, Pauwelsstr. 30, 52074 Aachen, Germany; 2 Department of Otorhinolaryngology, Oberhavel Kliniken, Marwitz Str. 91, 16761 Hennigsdorf, Germany; 3 Department of Oto-Rhino-Laryngology, Head and Neck Surgery, Würzburg University Hospital, Josef-Schneider-Str. 11, 97080 Würzburg, Germany

**Keywords:** chronic dizziness, DHI, long-term course

## Abstract

**Background:** Dizziness is a common symptom arising from disorders across multiple organ systems. Diagnostic pathways are often prolonged, and uncertainty may increase patients’ subjective burden. **Aims/Objectives:** This study describes the long-term course of functional impairment in patients with chronic, complex dizziness with delayed or inconclusive diagnostic classification using longitudinal DHI data. **Material and Methods:** This retrospective longitudinal study included patients presenting between 2009 and 2012 with persistent dizziness of at least one year and no definitive diagnosis at baseline. Initial DHI scores were compared with follow-up data collected 14–17 years later via structured telephone interviews. Changes in total and subdomain scores (functional, emotional, and physical) were analyzed, with subgroup analyses (sex, age, and diagnostic status). **Results:** Fifty-one patients were included. Median total DHI score decreased from 48.0 to 14.0 (Z = −6.13, *p* < 0.001). Median within-patient change was −28 (95% CI: −44 to −15). A total of 49/51 patients (96%) showed improvement. All DHI subdomains improved significantly, with the largest change observed in the functional domain (median −10, 95% CI: −15 to −4). Improvements were consistent across subgroups and largely independent of epidemiological and diagnostic factors. **Conclusions and Significance:** Patients with complex chronic dizziness show substantial long-term improvement in DHI scores. Further studies in larger cohorts are needed to determine whether a generalizable prognosis can be established.

## 1. Introduction

Dizziness is a very common symptom that can result from disorders affecting a wide variety of organ systems. Particularly in the chronic setting, dizziness frequently involves multifactorial or complex etiologies, with overlapping vestibular, neurological, and psychosomatic components [1]. This complexity often results in protracted diagnostic processes. A national survey conducted in Switzerland demonstrated that primary care physicians were unable to establish a diagnosis in 50% of patients presenting with dizziness [2]. Similar findings were reported in an emergency department cohort, where the etiology remained unclear in 61.97% of cases among patients with chronic vestibular symptoms [3]. Throughout the resulting extended diagnostic pathways, patients may receive multiple or evolving diagnoses over time. A diagnosis-specific study on Ménière’s disease further showed that 20% of patients experienced a diagnostic delay of more than five years [4].

Similarly to its underlying factors, the consequences of dizziness can be highly heterogeneous and mutually reinforcing across physical, functional, and psychosocial domains.

On a physical level, dizziness in older individuals is strongly associated with an elevated risk of falls [5]. Functionally, dizziness may lead to limitations in daily activities and reduced work performance [6]. Psychologically and emotionally, patients commonly experience anxiety, depressed mood, and social burden due to the persistent impact of their symptoms [6]. Indeed, a systematic review and meta-analysis reported a high prevalence of anxiety (31.4%) and depression (28.3%) among adults with vestibular disorders and found significant correlations between Dizziness Handicap Inventory (DHI) scores and anxiety/depression scales [7].

Psychological distress is strongly associated with perceived dizziness-related disability, with higher DHI scores correlating with symptoms of anxiety and depression across diverse vestibular conditions [8]. These findings underline that dizziness is not only a sensory or balance disorder but also a condition in which functional, emotional, and physical consequences interact and amplify one another, creating a self-reinforcing cycle of disability. Moreover, early psychological intervention has been shown to improve both emotional well-being and vestibular compensation [9]. Providing early prognostic information and timely psychological counseling may help to interrupt this cycle by reducing uncertainty and improving coping strategies.

In view of these observations, longitudinal data on diagnosis-independent long-term trajectories of functional impairment are needed to support the future development of prognostic frameworks capable of providing early, diagnosis-independent estimates of likely functional outcomes. Such approaches may ultimately facilitate earlier counseling and management before a definitive diagnosis is established.

To date, most studies in the literature have focused on outcomes stratified by specific diagnostic categories or diagnostic subgroups [10,11]. As previously outlined, establishing a diagnosis in chronic dizziness can be challenging. The often-prolonged absence of a definitive diagnosis—and thus of a clear prognosis—may contribute to an increased psychological burden in affected patients. The present study aims to evaluate the long-term course of functional limitations in patients with chronic, complex dizziness using the DHI. The resulting data are intended to serve as an exploratory foundation for future studies to assess whether a general, diagnosis-independent prognosis can be defined.

## 2. Materials and Methods

In the present study, we investigated the long-term course of complex chronic dizziness across diagnostic categories. To this end, we analyzed DHI questionnaires that had been completed by patients with chronic dizziness at a time when their diagnosis was unclear or insufficiently established. These baseline data were compared with findings from a structured follow-up telephone interview conducted 14 to 17 years later.

Changes in DHI scores over time were examined both at the level of the total score and according to the established DHI subdomains (functional, emotional, and physical). In addition, the longitudinal course was analyzed in relation to clinical and epidemiological patient characteristics.

### 2.1. Dizziness Handicap Inventory (DHI)

The DHI is a validated patient-reported outcome measure used to assess the perceived impact of dizziness on daily life across functional, emotional, and physical domains. It consists of 25 items with a total score ranging from 0 to 100, with higher scores indicating greater perceived handicap [12]. In the present study, a validated German version of the DHI was used [13].

### 2.2. Patient Selection

Data from patients who underwent a multi-day inpatient neuro-otological diagnostic workup in our department (including more than 10 different examinations) between 2009 and 2012 were analyzed. Eligibility was determined based on the clinical documentation available at the beginning of this diagnostic workup, when the DHI was first completed. Patients were included in the analysis if they met all of the following criteria:

1. Persistent dizziness symptoms for at least one year prior to presentation.
2. Previous evaluation by at least one otorhinolaryngology specialist without establishment of a definitive diagnosis, or with a diagnosis considered insufficient to adequately explain the patient’s symptoms.
3. No sufficient explanatory diagnosis established during the initial outpatient assessment at our clinic, resulting in the indication for an extended, multi-day inpatient diagnostic workup for chronic dizziness.

Upon completion of the inpatient neuro-otological diagnostic work-up and before discharge, all patients received a comprehensive physician consultation including education about the established diagnosis, whenever a definitive diagnosis could be made, as well as treatment recommendations according to the standard of care at the time. These included performance and instruction of canalith repositioning maneuvers for benign paroxysmal positional vertigo (BPPV), prescription of vestibular rehabilitation and sensorimotor training for unilateral or bilateral vestibulopathy, and a stepwise treatment approach for Ménière’s disease ranging from betahistine therapy to surgical interventions, as appropriate.

### 2.3. Patient Survey

After review of the inclusion criteria using patients’ medical records, 141 eligible patients were identified. Seventy-six patients could no longer be contacted, and six were deceased. Five patients declined participation, and in three cases the questionnaire could not be completed. In total, 51 patients completed the DHI questionnaire. Of these, 47 were completed as part of a structured telephone interview and four in written form.

To assess the representativeness of the 51 patients included in the follow-up analysis, baseline DHI scores were compared between the re-evaluated patients and the 90 patients lost to follow-up. No significant difference was observed between groups, with median baseline total DHI scores of 48 (42 to 60) and 50 (34.5 to 66) in the study cohort and excluded patients, respectively.

The duration of the telephone interviews ranged from 15 to 40 min. Each call began with a standardized explanation of the study framework, followed by a brief inquiry into the patient’s current health status, with particular emphasis on their dizziness symptoms. Subsequently, the 25 items of the DHI questionnaire were administered according to the prescribed format. All telephone interviews were conducted by the same investigator to ensure consistency.

### 2.4. Statistical Analysis

Epidemiological and clinical data were obtained from patients’ medical records. Diagnoses included in the analysis were reviewed and updated, if necessary, in accordance with current clinical guidelines for vestibular disorders [14].

Changes in DHI scores between baseline and follow-up were analyzed for the overall cohort, including total scores and the three subdomains (functional, emotional, and physical). Within-patient changes were assessed using the Wilcoxon signed-rank test.

For subgroup analyses, patients were stratified according to sex, age group, and diagnostic status. Comparisons between two independent groups (e.g., sex, presence vs. absence of diagnosis) were performed using the Mann–Whitney U test. Comparisons across more than two groups (e.g., age categories) were conducted using Kruskal–Wallis test. Holm’s correction was applied to account for multiple comparisons across subdomains. All tests were two-tailed, and *p*-values < 0.05 were considered statistically significant.

Statistical analyses were performed using SPSS (Version 11.0.0, IBM Corp., Armonk, NY, USA). Graphical visualizations were generated using GraphPad Prism (Version 29, GraphPad Software, San Diego, CA, USA).

### 2.5. Ethics Statement

This study was conducted in accordance with the principles of the Declaration of Helsinki. All participants provided written informed consent prior to inclusion in the study. Ethical approval was obtained from the Ethics Committee of RWTH Aachen University (approval number: K 25-432).

## 3. Results

### 3.1. Epidemiological Data

A total of 51 patients were included in the study, 27 male and 24 female. The mean age at initial presentation was 54.2 ± 12.7 years. In 30 patients, a peripheral vestibular diagnosis could be established in accordance with the criteria of the German Clinical Practice Guideline “Vestibular Function Disorders,” adequately explaining the patients’ symptomatology [14]. Twenty-one patients remained without a definitive peripheral vestibular diagnosis after the multi-day neuro-otological dizziness evaluation in our department or were assigned a diagnosis that did not sufficiently account for the overall clinical presentation of their symptoms. For detailed epidemiological data, see Table 1.

### 3.2. Overall Change in DHI Scores

Within the overall cohort, 49 patients showed a decrease in DHI scores between the initial assessment and follow-up, while one patient showed an increase and one patient had an unchanged DHI score.

**Figure 1:**
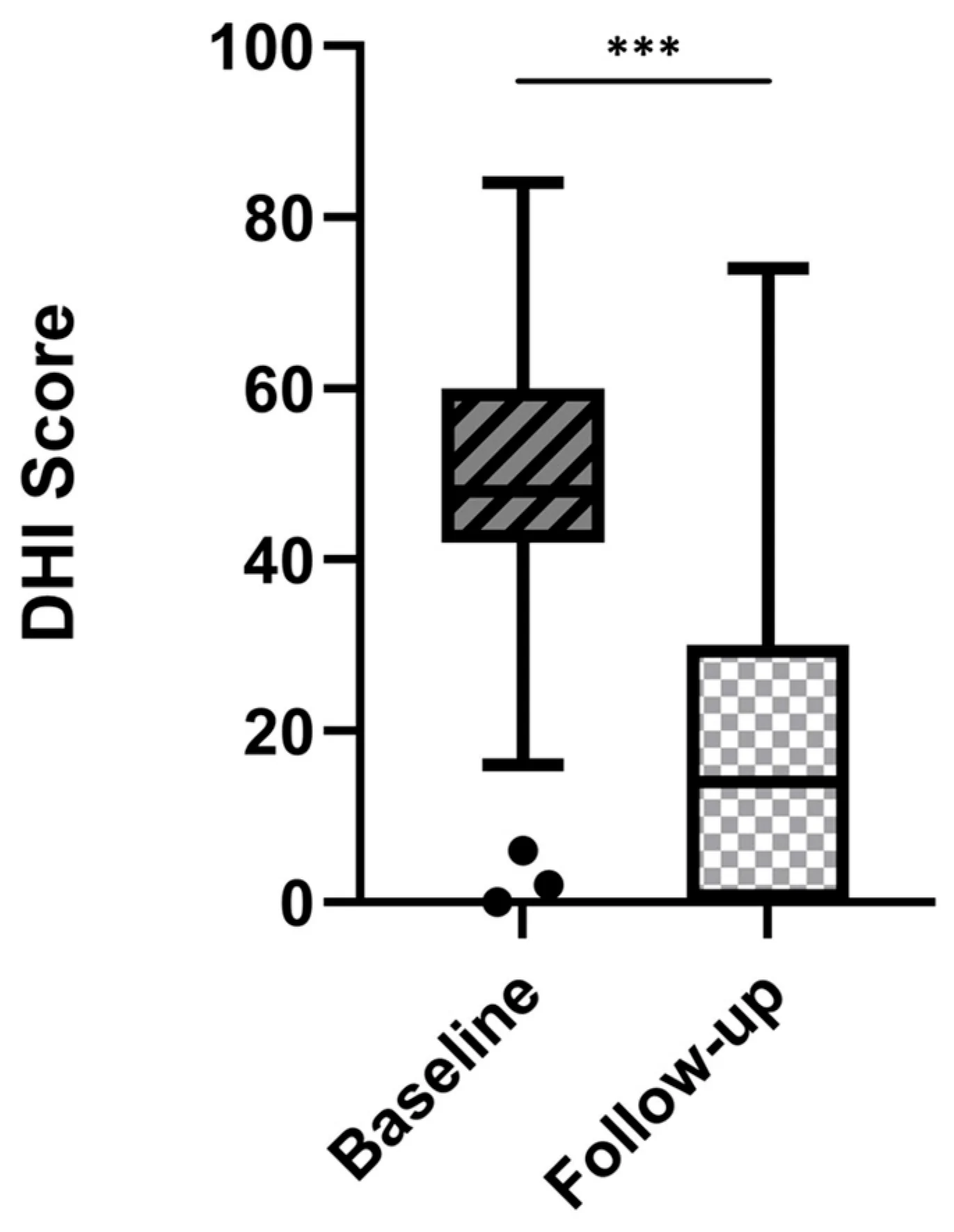
DHI total scores in the overall study cohort at baseline and follow-up, demonstrating a significant reduction over time. Boxes represent the interquartile range (IQR; 25th–75th percentile) with the median indicated as the central line; whiskers extend to 1.5 × IQR (Tukey definition); and individual data points beyond the whiskers are displayed as outliers. Significance levels are indicated as *** *p* < 0.001.

The median total DHI score decreased from 48.0 at baseline to 14.0 at follow-up. The mean change in DHI score per patient was −29.73, and the median within-patient change was −28, indicating a statistically significant reduction (Z = −6.13, *p* < 0.001). Overall, DHI scores across the cohort indicate a marked improvement in symptom burden and a substantial reduction in dizziness-related handicap over time (Figure 1).

**Figure 2:**
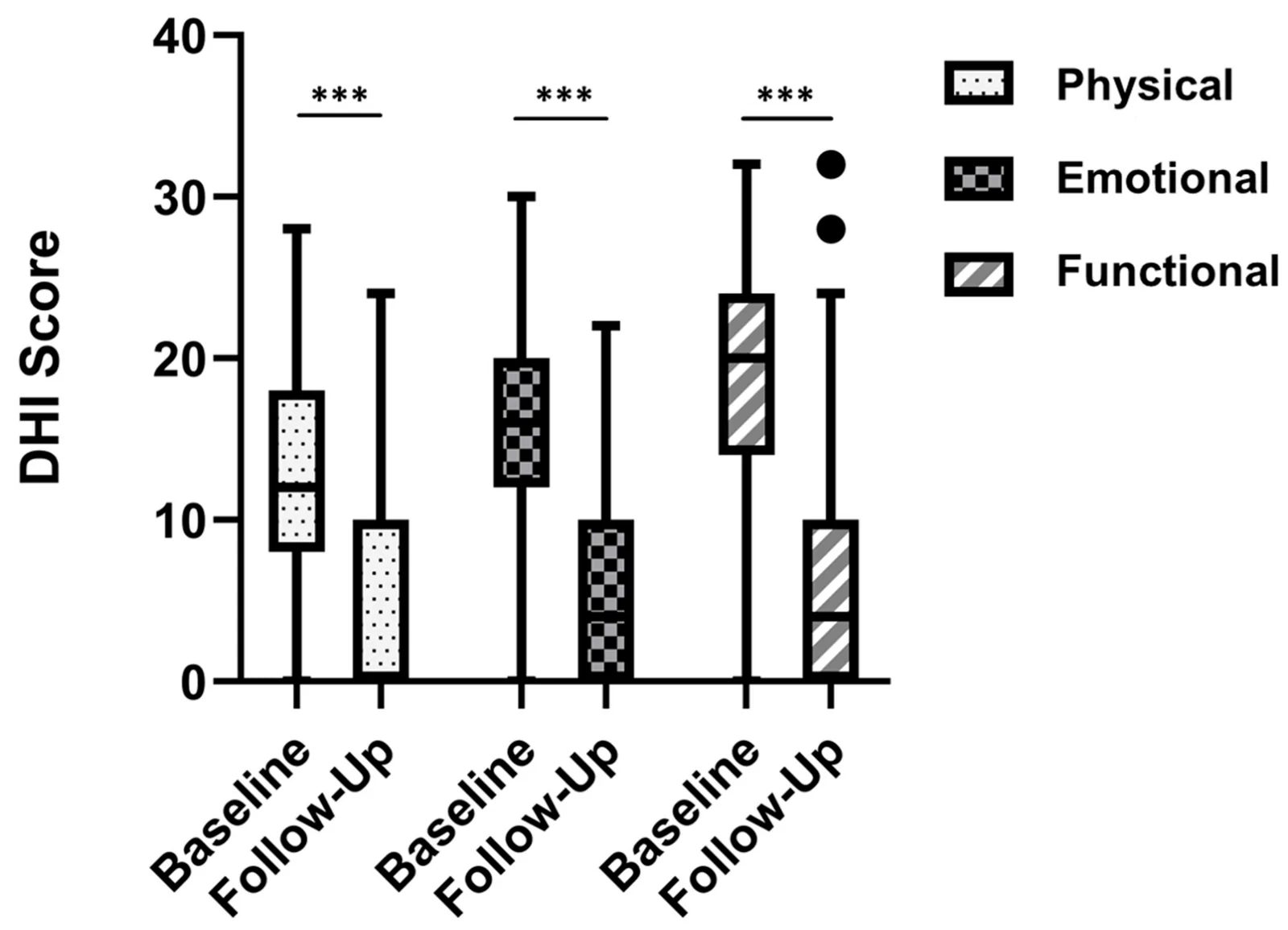
DHI subscale scores (physical, functional, and emotional) in the overall study cohort at baseline and follow-up, showing significant reductions across all subdomains. Boxes represent the interquartile range (IQR; 25th–75th percentile) with the median indicated as the central line; whiskers extend to 1.5 × IQR (Tukey definition); and individual data points beyond the whiskers are displayed as outliers. Significance levels are indicated as *** *p* < 0.001.

Considering the subdomains, the most pronounced reduction in DHI scores was observed in the functional domain, followed by the emotional and, lastly, the physical domain (mean change: −11.76 vs. −10.24 vs. −7.73). The decrease from baseline to follow-up was statistically significant for each subdomain (functional: Z = −6.6, *p* < 0.001; emotional: Z = −5.68, *p* < 0.001; physical: Z = 6.25, *p* < 0.001). In pairwise comparisons between domains, the reduction in the functional domain was significantly greater than in the physical domain (*p* = 0.03), whereas no statistically significant differences were observed between the remaining domains (Table 2, Figure 2).

### 3.3. Change in DHI Score Stratified by Epidemiological Characteristics

For further analysis, the cohort was stratified according to selected epidemiological factors. When evaluating DHI score changes by sex, female patients showed a mean within-patient change of −36.42 points, whereas male patients exhibited a decrease of −23.78 points. In both groups, the reduction from baseline to follow-up was statistically significant (Z = −4.25, *p* < 0.001; Z = −4.46, *p* < 0.001). In the direct comparison between groups, the decrease in DHI scores was significantly greater in female patients than in male patients (*p* = 0.04) (Table 3).

The cohort was additionally stratified by age into three groups (<46, 46–60, and >60 years). Within each age group, a statistically significant reduction in DHI scores from baseline to follow-up was observed, with mean within-patient changes of −24.2, −32.1, and −30.53, respectively (Z = −2.67, *p* = 0.008; Z = −3.97, *p* < 0.001; Z = −3.92, *p* < 0.001). However, no statistically significant differences were found between the age groups (Table 4).

Finally, patients were stratified according to whether a peripheral vestibular diagnosis could be established during the multi-day neuro-otological assessment in our department that sufficiently explained the patients’ symptoms. A statistically significant improvement in DHI scores was observed in both groups, with mean within-patient changes of −30.07 in patients with a diagnosis and −29.24 in patients without a diagnosis (Z = −4.71, *p* < 0.001; Z = −3.96, *p* < 0.001). No statistically significant difference was found between the two groups (Table 5).

## 4. Discussion

The clinical relevance of an early, approximate, and diagnosis-independent understanding of long-term outcomes in patients with complex dizziness is particularly high, given the often-substantial subjective burden experienced by this patient population [6,7,8]. In this context, the present study was conducted as an exploratory analysis intended as a first step toward characterizing long-term trajectories of functional impairment in this patient population within a long-term observational framework.

In the present study, we investigated the long-term course of impairment in patients with complex dizziness in whom either a diagnosis could only be established after considerable delay and extensive diagnostic work-up, or no definitive diagnosis could be made. Our analysis demonstrated a clear trend toward significant improvement over the long-term follow-up period. This improvement was observed across all three subcategories of the DHI and was consistent across subgroups stratified by sex, age, and diagnostic status.

To contextualize these findings within the existing literature, studies investigating diagnosis-specific courses of vestibular disorders may be considered. In line with our results, a large Dizziness and Vertigo Registry study including patients with various specific diagnoses (e.g., phobic postural vertigo, benign paroxysmal positional vertigo, and unilateral vestibulopathy) reported an improvement in DHI scores in 72% of patients after two years [10]. Similarly, in patients with primary somatoform vertigo and dizziness, significant improvements in patient-reported outcomes across several standardized questionnaires were observed after three years [15]. In a longitudinal follow-up study of patients with Ménière’s disease, vertigo symptoms ceased in approximately 50% of patients after 14 years [11]. Furthermore, several studies evaluating DHI outcomes in patients with Ménière’s disease before and after specific conservative or surgical interventions have demonstrated significant improvements [16,17,18]. Although the reported endpoints are heterogeneous and the overall body of literature on long-term outcomes in vestibular disorders remains limited, an overall trend toward symptom improvement across different diagnoses and time frames is evident. Against this background, the present findings are consistent with previously reported observations and may serve as an exploratory contribution to the understanding of long-term trajectories in this patient population. The clinical relevance of an early prognostic perspective remains of interest, but cannot be conclusively addressed based on the present study design. Future studies with larger cohorts, prospective designs, and varying observation periods, including intermediate follow-up intervals of 1–2 years, may help to further characterize the temporal course of functional impairment and evaluate the potential for clinically meaningful prognostic approaches, while also accounting for potential influences such as selection effects, natural recovery, treatment exposure, and regression to the mean. In addition, incorporating complementary patient-reported and functional outcome measures, such as work ability, return-to-work, health-related quality of life, and healthcare utilization, may provide a more comprehensive assessment of the long-term clinical and socioeconomic impact of chronic dizziness.

To further characterize the observed long-term changes, additional analyses examined domain-specific patterns within the DHI as well as potential subgroup differences across demographic and diagnostic factors.

Analysis of the DHI subcategories showed differential changes across domains, with greater improvements observed in the functional and emotional subscales compared to the physical subscale, although only the difference in the functional domain reached statistical significance. While this pattern describes domain-specific differences in symptom change over time, it does not allow for conclusions regarding underlying mechanisms. In particular, it remains unclear whether these findings reflect true changes in symptom burden, adaptation processes, or differences in symptom perception over time. Similarly, the absence of a significant association between diagnostic status and the trajectory of DHI scores suggests that long-term changes were not strongly driven by diagnosis-specific factors. However, this finding should be interpreted cautiously, as it is based solely on questionnaire-derived outcomes without objective clinical or vestibular measurements.

Accordingly, subgroup analyses based on epidemiological factors should be interpreted as exploratory. These analyses were not the primary objective of the study, and subgroup sizes were heterogeneous and not adjusted for potential confounders such as comorbidities or baseline DHI scores. Several limitations of the present study should be considered. The retrospective design and the very long follow-up period introduce an inherent risk of selection bias, as only 51 of the 141 initially eligible patients were available for analysis, and patients with persistent or worsening symptoms may be underrepresented due to loss to follow-up.

Outcome assessment was conducted via telephone-based administration of the Dizziness Handicap Inventory, resulting in exclusively patient-reported data without accompanying objective clinical or vestibular measures. This limits the ability to relate perceived functional impairment to objectively verifiable deficits.

Patient-specific factors such as pre-existing comorbidities and therapeutic interventions received over the extended observation period were not analyzed in detail. As these factors may influence symptom perception, functional impairment, and quality of life, their potential contribution to the observed long-term changes in DHI scores remains unclear. Furthermore, treatment exposure was neither controlled nor systematically recorded. Although all patients received diagnosis-based treatment recommendations at discharge according to the standard of care at the time, adherence to these recommendations and the actual implementation of the proposed therapies during follow-up were not assessed. Consequently, the specific contribution of therapeutic interventions to the observed improvement cannot be determined. In addition, regression to the mean may have contributed to the observed results.

In this context, the observed sex-related differences in DHI improvement should also be interpreted with caution. Given that the existing literature suggests sex-specific differences in the prevalence and manifestation of common vestibular disorders, these findings may, at least in part, reflect underlying differences in diagnostic distribution rather than true sex-specific effects [19].

It should also be noted that the study cohort reflects clinical diagnostic practice between 2009 and 2012. Diagnostic concepts that are routinely applied today, including PPPD and contemporary Bárány criteria for vestibular migraine, were not yet fully established during the recruitment period [20,21]. Consequently, some patients who remained without a definitive diagnosis in the present analysis might today receive a more specific diagnostic classification.

Taken together, these limitations underscore the need for further research to better elucidate influencing factors, including potential sex-specific effects. For a more detailed evaluation and to enable refined prognostic modeling, future studies with larger sample sizes and, ideally, prospective study designs are warranted. It should also be noted that two patients in the present cohort did not show improvement in DHI scores over time. Due to the limited sample size, further characterization of these individual trajectories was beyond the scope of the present analysis. Nevertheless, the identification of factors associated with persistent impairment or absent improvement may represent an important objective for future studies in larger cohorts.

## 5. Conclusions

In summary, among patients with complex dizziness who responded to very long-term follow-up, DHI scores were lower compared to baseline, indicating a significant improvement over time. Further prospective studies are needed to confirm these observations and to clarify whether robust, diagnosis-independent prognostic models can be developed. In this context, intermediate follow-up periods (e.g., 1–2 years) may be particularly useful to better characterize the temporal dynamics of functional impairment.

## Figures and Tables

**Table 1:** Demographic and neuro-otological clinical characteristics of the study cohort. The category headings are shown in bold.

**Sex Distribution**	
Female	24 (47.06%)
Male	27 (52.94%)
**Age Distribution**	
<46 Years	10 (19.61%)
46–60 Years	21 (41.18%)
>60 Years	20 (39.22%)
**Diagnosis in Multi-Day Neuro-Otologic Workup**	
Diagnosed Patients	30 (58.82%)
Undiagnosed Patients	21 (41.18%)
Unilateral Ménière’s disease	9
Bilateral Ménière’s disease	3
Unilateral peripheral vestibulopathy (not fully compensated)	11
bilateral peripheral vestibulopathy	1
Benign paroxysmal positional vertigo	6

**Table 2:** Change in Dizziness Handicap Inventory scores in the overall cohort and across subcategories. A significant reduction is observed both overall and in all three subcategories.

	Overall		Physical		Emotional		Functional	
	Baseline	Follow-up	Baseline	Follow-up	Baseline	Follow-up	Baseline	Follow-up
Mean ± SD	47.22 ± 19.02	17.49 ± 19.46	12.94 ± 6.52	5.22 ± 6.51	18.47 ± 8.17	6.71 ±7.89	15.8 ± 7.18	5.57 ± 6.64
Median (IQR)	48 (42 to 60)	14 (0 to 30)	12 (9 to 18)	2 (0 to 10)	20 (14 to 24)	4 (0 to 10)	16 (12 to 20)	4 (0 to 10)
Mean ΔDHI (within-patient change)	−29.73 ± 20.39		−7.73 ± 6.96		−11.76 ± 8.48		−10.24 ± 7.93	
Median ΔDHI	−28 (−44 to −15)		−8.0 (−12 to −4)		−12 (−18 to −6)		−10 (−15 to −4)	

**Table 3:** Change in DHI scores stratified by sex. A significant reduction in scores is observed in both male and female patients.

	Female		Male	
Assessment Timepoint	Baseline	Follow-up	Baseline	Follow-up
Mean ± SD	53.59 ± 15.6	17.17 ± 22.62	41.56 ± 19.97	17.78 ± 16.13
Median (IQR)	51 (42 to 64)	2 (0 to 33.5)	44 (31 to 56)	18 (2 to 27)
Mean ΔDHI (within-patient change)	−36.42 ± 20.74		−23.78 ± 18.11	
Median ΔDHI (IQR)	−42 (−47 to −17.5)		−18 (−35 to −13)	

**Table 4:** Change in DHI scores stratified by age groups. Scores show a significant decrease in all three groups.

	<46 Years		46–60 Years		>60 Years	
Assessment Time-point	Baseline	Follow-up	Baseline	Follow-up	Baseline	Follow-up
Mean ± SD	36.6 ± 24.43	12.4 ± 19.55	49.33 ± 15.41	17.23 ± 17.07	50.3 ± 17.44	20.3 ± 21.16
Median (IQR)	44 (16.5 to 53.5)	0 (0 to 15)	48 (42 to 60)	14 (2 to 30)	50 (42 to 60.5)	18 (0 to 27)
Mean ΔDHI (within-patient change)	−24.2 ± 21.12		−32.1 ± 19.12		−30.53 ± 21.19	
Median ΔDHI (IQR)	−15 (−45 to −4.5)		−32 (−44 to −18)		−30 (−44 to −14)	

**Table 5:** Change in DHI scores stratified by the presence or absence of a peripheral vestibular diagnosis established during the multi-day neuro-otological assessment. Significant improvement is observed in both groups.

	Diagnosed Patients		Undiagnosed Patients	
Assessment Timepoint	Baseline	Follow-up	Baseline	Follow-up
Mean ± SD	46.8 ± 21.19	13.83 ± 28.79	47.81 ± 15.38	18.57 ± 20.33
Median (IQR)	45 (42–60)	12 (0–29)	48 (42 to 56)	14 (0 to 30)
Mean ΔDHI (within-patient change)	−30.07 ± 21		−29.24 ± 19.46	
Median ΔDHI (IQR)	−30 (−14.5 to −45.5)		−28 (−16 to −42)	

## Data Availability

All data are available on request from the corresponding author.

## References

[1] Münst J., Pudszuhn A., V. Bernstorff M., Obermueller T., Erdur H., Audebert H.J., Rose M., Reisshauer A., Hoffmann I., Schönfeld U. (2022). Unklare chronische Schwindelsyndrome—Erfahrungen mit einem interdisziplinären stationären Diagnostikkonzept [Unclear chronic vertigo syndromes-experiences with an interdisciplinary inpatient diagnostic concept]. HNO.

[2] Zwergal A., Mantokoudis G., Heg D., Kerkeni H., Diener S., Kalla R., Korda A., Candreia C., Welge-Lüssen A., Tarnutzer A.A. (2023). What is the current status of primary care in the diagnosis and treatment of patients with vertigo and dizziness in Switzerland? A national survey. Front. Neurol..

[3] Comolli L., Korda A., Zamaro E., Wagner F., Sauter T.C., Caversaccio M.D., Nikles F., Jung S., Mantokoudis G. (2023). Vestibular syndromes, diagnosis and diagnostic errors in patients with dizziness presenting to the emergency department: A cross-sectional study. BMJ Open.

[4] Pyykkö I., Zou J., Vetkas N. (2024). Changes in symptom pattern in Meniere’s disease by duration: The need for comprehensive management. Front. Neurol..

[5] Li Y., Smith R.M., Whitney S.L., Seemungal B.M., Ellmers T.J. (2024). Association between dizziness and future falls and fall-related injuries in older adults: A systematic review and meta-analysis. Age Ageing.

[6] Jang Y., Hur H.J., Park B., Park H.Y. (2024). Psychosocial Factors Associated with dizziness and chronic dizziness: A nationwide cross-sectional study. BMC Psychiatry.

[7] Kim C.H., McCray L.R., Nguyen S.A., Staab J.P., Jafri S., Rizk H. (2025). Anxiety and Depression in Adults with Vestibular Disorders: A Systematic Review and Meta-Analysis. Laryngoscope.

[8] Hong S.M., Lee H.J., Lee B., Park S., Hong S.K., Park I., Kim Y.B., Kim H. (2013). Influence of vestibular disease on psychological distress: A multicenter study. Otolaryngol. Head Neck Surg..

[9] Peng F., Mei R., Liu C., Liu X., Xiong J., Lv L., Wang F. (2022). Health Promotion Combined with Psychological Care Improves Vestibular Function in Patients with Vestibular Neuritis. Contrast Media Mol. Imaging.

[10] Obermann M., Bock E., Sabev N., Lehmann N., Weber R., Gerwig M., Frings M., Arweiler-Harbeck D., Lang S., Diener H.-C. (2015). Long-term outcome of vertigo and dizziness associated disorders following treatment in specialized tertiary care: The Dizziness and Vertigo Registry (DiVeR) Study. J. Neurol..

[11] Green J.D., Blum D.J., Harner S.G. (1991). Longitudinal followup of patients with Menière’s disease. Otolaryngol. Head Neck Surg..

[12] Jacobson G.P., Newman C.W. (1990). The development of the Dizziness Handicap Inventory. Arch. Otolaryngol. Head Neck Surg..

[13] Volz-Sidiropoulou E., Takahama J., Gauggel S., Westhofen M. (2010). Das “Dizziness Handicap Inventory”: Erste psychometrische Kennwerte einer Deutschen Version [The ‘dizziness handicap inventory’: Initial psychometric evaluation of the German version]. Laryngorhinootologie.

[14] Arbeitsgemeinschaft der Wissenschaftlichen Medizinischen Fachgesellschaften (AWMF) Guideline: Vestibular Function Disorders. AWMF Guideline No. 017/078. Version 2021, Valid Until 2026. https://www.awmf.org/leitlinien/detail/ll/017-078.html.

[15] Tschan R., Best C., Wiltink J., Beutel M.E., Dieterich M., Eckhardt-Henn A. (2013). Persistence of symptoms in primary somatoform vertigo and dizziness: A disorder “lost” in health care?. J. Nerv. Ment. Dis..

[16] Socher D.D., Socher J.A., Azzi V.J. (2012). Evaluation of quality of life pre- and post-vestibular rehabilitation in patients with benign paroxysmal positional vertigo associated with Meniere’s disease. Int. Arch. Otorhinolaryngol..

[17] Zhong J., Li X., Xu J., Chen W., Gao J., Lu X., Liang S., Guo Z., Lu M., Li Y. (2023). Analysis of cognitive function and its related factors after treatment in Meniere’s disease. Front. Neurosci..

[18] Zhang D., Li X., Van de Berg R., Lyu Y., Dayimu A., Song Y., Duan M., Jian H., Li Y., Wang J. (2025). A Trial of Semicircular Canal Plugging vs. Intratympanic Gentamicin for Menière’s Disease. Laryngoscope.

[19] Schifino E., Joffily L., Koohi N., Kaski D. (2025). Sex differences in dizziness diagnoses across acute and chronic neurological settings. Neurol. Sci..

[20] Staab J.P., Eckhardt-Henn A., Horii A., Jacob R., Strupp M., Brandt T., Bronstein A. (2017). Diagnostic criteria for persistent postural-perceptual dizziness (PPPD): Consensus document of the committee for the Classification of Vestibular Disorders of the Bárány Society. J. Vestib. Res..

[21] Lempert T., Olesen J., Furman J., Waterston J., Seemungal B., Carey J., Bisdorff A., Versino M., Evers S., Newman-Toker D. (2012). Vestibular migraine: Diagnostic criteria. J. Vestib. Res..

